# Venetoclax for Treating Chronic Lymphocytic Leukaemia: An Evidence Review Group Perspective of a NICE Single Technology Appraisal

**DOI:** 10.1007/s40273-017-0599-9

**Published:** 2017-12-08

**Authors:** Hema Mistry, Chidozie Nduka, Martin Connock, Jill Colquitt, Theodoros Mantopoulos, Emma Loveman, Renata Walewska, James Mason

**Affiliations:** 1 Warwick Evidence, Warwick Medical School, University of Warwick, Gibbet Hill Road, Coventry, CV4 7AL UK; 2 Effective Evidence LLP, 26 The Curve, Waterlooville, Hampshire PO8 9SE UK; 3 The Royal Bournemouth Hospital NHS Foundation Trust, Castle Lane East, Bournemouth, BH7 7DW UK

## Abstract

Venetoclax is licensed to treat relapsed or refractory (R/R) chronic lymphocytic leukaemia (CLL). As part of the Single Technology Appraisal (STA) ID944, the National Institute for Health and Care Excellence (NICE) invited AbbVie, the manufacturer, to submit evidence on the use of venetoclax, within its licensed indication. The Evidence Review Group (ERG), Warwick Evidence, was asked to provide an independent and critical review of the submitted evidence. Evidence came from three single-arm trials in CLL patients with or without 17p deletion [del(17p])/TP53 chromosomal abnormalities. The anticipated licensed indication specified that venetoclax-eligible del(17p)/TP53 patients should have not responded to, or be deemed unsuitable for, B-cell receptor inhibitor (BCRi) therapy, and that non-del(17p)/TP53 patients should have not responded to both chemoimmunotherapy and BCRi therapy. The three trials were heterogeneous in terms of both del(17p)/TP53 status and previous exposure to BCRi therapy. The M13-982 study investigated 158 R/R CLL patients with the 17p deletion, but only a small number had received previous BCRi therapy; the M12-175 study investigated 67 patients with CLL or small lymphocytic lymphoma, some with the 17p deletion, but very few previously treated with BCRi therapy; and the M14-032 study included 105 patients previously treated with BCRi therapy (either idelalisib or ibrutinib), some of whom had unknown mutation status. The ERG concluded that the study populations did not directly conform to those specified in the licensed indication or in the NICE scope. Outcomes reported included overall response rate (ORR), duration of response, progression-free survival (PFS) and overall survival (OS); adverse events were reported for the pooled population of all three studies, as well as separately for each study. The median PFS was 41.4 and 27.2 months among patients in the M12-175 and M13-982 trials, respectively, whereas the median PFS was not reached in the M14-032 trial. Some results were designated academic in confidence and cannot be reported here. The submission provided a de novo partitioned survival cost-effectiveness model with three health states: pre-progression, post-progression and dead. Transition probabilities between health states were estimated using Weibull models for PFS and OS. The ERG judged the model structure to be appropriate. Venetoclax was compared with best supportive care (BSC) in patients with or without del(17p)/TP53 mutation status, and with palliative care (PC). To populate the del(17p)/TP53 venetoclax arm, the submission pooled del(17p)/TP53 patients from all three studies and fitted Weibull models for PFS and OS. PFS and OS models for non-del(17p)/TP53 venetoclax patients were obtained by applying hazard ratios (HRs) to the del(17p)/TP53 OS and PFS models, derived using Cox’s regression analysis comparing del(17p)/TP53 and non-del(17p)/TP53 patients pooled from the M14-032 and M12-175 studies. The ERG expressed reservations about the company’s pooling procedure, but acknowledged its expedience given the small evidence base. For the BSC comparator arm, the submission used the rituximab + placebo arm from a randomised controlled trial comparing idelalisib + rituximab versus placebo + rituximab (‘study 116’). Weibull regression data for OS and PFS were taken from the idelalisib STA (ID764) submitted by Gilead to NICE. The ERG considered the use of the study 116 rituximab arm to be inconsistent with the licensed indication for venetoclax because these patients had neither not responded to nor were inappropriate for BCRi therapy, being eligible to be randomised to idelalisib. Another difficulty was the requirement for a technical correction in survival analysis because of considerable switching from rituximab to idelalisib. The ERG considered that post-progression survival of patients from the idelalisib arm of study 116 provided a more appropriate representation of BSC since these patients had not responded to BCRi therapy, consistent with venetoclax’s licensed indication. For PC, the company submission used data from the UK CLL Forum. The company’s base-case analysis indicated that venetoclax was clinically effective, but the resulting incremental cost-effectiveness ratios (ICERs) for del(17p)/TP53 (£39,940/quality-adjusted life-year [QALY] gained) and non-del(17p)/TP53 (£47,370/QALY gained) patients were well above the NICE threshold of £20,000–30,000/QALY. The ERG identified two errors in the implementation of the company’s parametric models—one related to the implementation of HRs, and the other to the derivation of the Weibull shape parameters obtained from the Gilead idelalisib submission. The ERG made plausible adjustments to the company’s base-case and corrected errors, resulting in a reduced estimate of the cost effectiveness of venetoclax in non-del(17p)/TP53 and del(17p)/TP53 indications; in the ERG’s preferred base case, using post-progression survival of patients in the idelalisib arm of study 116 as the BSC comparator, deterministic ICERs were higher than the company’s base-case for both indications: £57,476/QALY gained for del(17p)/TP53 and £77,779/QALY gained for non-del(17p)/TP53. The NICE Appraisal Committee’s preliminary recommendation was that venetoclax used within its licensed indication should not be recommended for use in the National Health Service (NHS). In response to the preliminary recommendation, the company submitted new analyses; however, at a subsequent appraisal committee meeting, the original recommendation was upheld and the committee concluded there were large uncertainties around the clinical effectiveness of venetoclax and BSC, and that under the committee’s preferred assumptions, the ICERs were higher than those generally considered cost effective, even when end-of-life criteria were taken into account. The company submitted further evidence, and the final guidance recommended venetoclax for use with the Cancer Drugs Fund for the two populations in this technology appraisal.

**Electronic supplementary material:**

The online version of this article (10.1007/s40273-017-0599-9) contains supplementary material, which is available to authorized users.

## Key Points for Decision Makers

**Table:** 

Identifying credible comparisons for single-arm (uncontrolled) trials presents considerable challenges to the National Institute for Health and Care Excellence (NICE) appraisal process, particularly when naive comparisons are presented.
Given the uncertainties inherent in uncontrolled comparisons and the potential for bias, the appraisal process can be expected to closely scrutinise comparisons selected for company submissions.
Venetoclax has been recommended for use within the Cancer Drugs Fund as an option for treating chronic lymphocytic leukaemia for patients with a 17p deletion/TP53 mutation who have not responded to, or have been deemed unsuitable for, a B-cell receptor pathway inhibitor, or without a 17p deletion/TP53 mutation who have not responded to both chemoimmunotherapy and B-cell receptor pathway inhibitor therapy.

## Introduction

The National Institute for Health and Care Excellence (NICE) is an independent body that appraises existing and new medical interventions, issuing guidance on their use within the National Health Service (NHS). Historically, NICE has assessed both the clinical and economic evidence within either a single technology appraisal (STA), for a single technology in a single indication, or in a multiple technology appraisal (MTA), for more than one technology or for one technology in more than one indication [1]. Latterly, NICE has relied on the STA process, with some STAs becoming more complex as a consequence, in terms of numbers of comparisons or indications. This paper summarises AbbVie’s clinical effectiveness and cost-effectiveness submission for use of its product venetoclax, within its licensed indication, for the treatment of chronic lymphocytic leukaemia (CLL), together with a description of the critique of the submitted evidence undertaken by the Evidence Review Group (ERG) appointed for this STA (Warwick Evidence), and a brief resume of the development of NICE guidance.

## Decision Problem

CLL manifests as the progressive accumulation of lymphocytes in the blood, bone marrow and lymphatic tissue. According to the CLL International Prognostic Index (CLL-IPI) published in 2016 [2], five major independent risk factors impact on the survival of patients following a diagnosis of CLL. The CLL-IPI scores these risk factors as follows: 17p deletion [del(17p])/TP53 (deleted/mutated) = 4; immunoglobulin heavy-chain variable-region (IGVH) not mutated = 2; β2-microglobulin > 3.5 mg/L = 2; clinical stage (Rai I–VI or Binet B–C) = 1; and age > 65 years = 1. To estimate an individual’s prognostic risk, the scores are summed; summed scores of 0–1 are categorised as low risk, 2–3 as intermediate risk, 4–6 as high risk, and 7–10 as very high risk.

Venetoclax is an orally administered inhibitor of B-cell lymphoma-2 (Bcl-2), an anti-apoptotic protein overexpressed in approximately 95% of CLL patients. Together with B-cell receptor (BCR) signalling, targeted by BCR inhibitor (BCRi) drugs such as idelalisib and ibrutinib, Bcl-2 represents an important element in CLL pathogenesis. The NICE scope for this STA requested clinical and cost-effectiveness evidence for venetoclax, within its licensed indication, compared with standard therapy without venetoclax. The anticipated marketing license specified two indications for venetoclax in CLL:

1. for the treatment of CLL in the presence of the del(17p) or TP53 mutation in adult patients who are unsuitable for or have not responded to a BCRi; and
2. for the treatment of CLL in the absence of the del(17p) or TP53 mutation in adult patients who have not responded to both chemoimmunotherapy and a BCRi.

## Submitted Evidence and Evidence Review Group (ERG) Critique

### Clinical Evidence

The company conducted a literature search and systematic review that yielded three uncontrolled studies providing relevant outcome and safety evidence for CLL patients who received venetoclax. These were single-arm, company-sponsored trials identified as M13-982 [3] (a multicentre international study), M14-032 [4] (a multicentre US study) and M12-175 [5] (a multicentre dose-ranging safety assessment study in the US and Australia). The three study populations were similar in terms of sex, ethnicity and Eastern Cooperative Oncology Group (ECOG) performance status, but they differed on age, del(17p)/TP53 chromosomal aberration status, and prior BCRi therapy.

Study M13-982 was a single-arm study in 158 relapsed/refractory (R/R) CLL patients with the del(17p)/TP53 chromosomal abnormality; a small proportion had received previous BCRi therapy, the proportion of patients unsuitable for BCRi was not reported, and a small number of patients were treatment-naive [3]. Study M14-032 was a two-group uncontrolled study of venetoclax in 105 patients after they had received previous BCRi therapy; the two groups comprised patients who had received ibrutinib and idelalisib, respectively [4]. Study M14-032 included similar proportions of patients with and without del(17p)/TP53 aberration status, most of whom had received prior chemoimmunotherapy. Study M12-175 evaluated the safety and efficacy of venetoclax in 67 patients with R/R CLL or small lymphocytic lymphoma (SLL); a substantial majority of patients had no previous BCRi therapy. This study included a mix of patients with and without the del(17p)/TP53 aberration [5]. The evidence is largely based on patient populations outside the UK, and the generalisability to the UK population is unclear. Table 1 summarises the baseline characteristics of the three studies, as presented in the company’s submission. None of the trials reported disease stage at study entry.

**Table 1:** Summary of key baseline characteristics of venetoclax studies

	Main cohort [*n* = 107]	Safety expansion [*n* = 51]	Ibrutinib failure [*n* = 43]	Idelalisib failure [*n* = 21]	Safety expansion [*n* = 41]	400 mg analysis set [*n* = 67]
Males	70 (65.4)	29 (56.9)	33 (76.7)	15 (71.4)	27 (65.9)	52 (77.6)
White	103 (97.2)	49 (96.1)	40 (93.0)	19 (90.5)	39 (95.1)	NR
Age ≥ 65 years	61 (57.0)	31 (60.8)	26 (60.5)	15 (71.4)	18 (43.9)	35 (52.2)
17p deletion						
Present	72 (67.3)	44 (86.3)	21 (48.8)	2 (9.5)	20 (48.8)	14 (20.9)
Absent	2 (1.9)	1 (2.0)	NR	NR	NR	40 (59.7)
Indeterminate	6 (5.6)	1 (2.0)	NR	NR	NR	4 (6.0)
TP53 mutation						
Present	61 (57.0)	32 (62.8)	15 (34.9)	1 (4.8)	11 (26.8)	14 (20.9)
Absent	17 (15.9)	9 (17.7)	NR	NR	NR	37 (55.2)
Indeterminate	6 (5.6)	1 (2.0)	NR	NR	NR	0
IGVH status						
Present	8 (7.5)	5 (9.8)	4 (9.3)	2 (9.5)	11 (26.8)	NR
Absent	28 (26.2)	17 (33.3)	NR	NR	NR	NR
Binet stage at diagnosis						
A	35 (32.7)	10 (19.6)	0 (0)	2 (9.5)	0 (0)	27 (40.3)
B	24 (22.4)	5 (9.8)	0 (0)	4 (19.1)	1 (2.4)	4 (6.0)
C	18 (16.8)	3 (5.9)	1 (2.3)	0 (0)	1 (2.4)	5 (7.5)
ECOG score						
0	42 (39.3)	NR	13 (30.2)	5 (23.8)	16 (39.0)	31 (46.2)
1	56 (52.3)	NR	27 (62.8)	14 (66.7)	22 (53.7)	34 (50.8)
2	9 (8.4)	NR	3 (7.0)	2 (9.5)	3 (7.3)	0

Data are expressed as *n* (%)

Percentages were calculated by the ERG using the sample size as the denominator; where percentages do not total 100, this is due to missing data (although the ERG notes that by including missing data, the total sample size for staging in M14 is 109 rather than 105); percentages in the company submission were calculated using non-missing data

*NR* not reported, *ECOG* Eastern Cooperative Oncology Group, *ERG* Evidence Review Group, *IGVH* immunoglobulin heavy-chain variable-region

In all three studies, venetoclax was administered orally and once daily in a stepwise weekly dose ramp-up schedule over 5 weeks to reach 400 mg daily, followed by 400 mg daily until disease progression or unacceptable toxicity.

Overall response rate was the primary outcome in studies M13-982 and M14-328, and a secondary outcome in study M12-175; safety/adverse effects was the primary outcome in study M12-175 and a secondary outcome in studies M13-982 and M14-328; and overall survival (OS) and progression-free survival (PFS) were additional secondary outcomes in all three studies. Health-related quality of life was measured in studies M13-982 and M14-382, but not in study M12-175.

The company’s submission presented a trial-by-trial narrative description of outcome results. The median PFS was 41.4 and 27.2 months among patients in the M12-175 and M13-982 trials, respectively, whereas the median PFS was not reached in the M14-032 trial. In addition, 86.5% of patients in the M13-982 trial, and 88.1% of patients in the prior ibrutinib arm and 95.2% of patients in the prior idelalisib arm in the M14-032 trial, were alive after 12 months. The other results were designated academic in confidence (AIC) or commercial in confidence (CIC) and cannot be reported here. A number of outcomes of relevance to the decision problem were not fully reported in the company submission for some studies (e.g. time to progression [TTP], European Organisation for Research and Treatment of Cancer [EORTC] QLQ-C30, EORTC QLQ-CLL16, and EuroQoL 5 dimensions 5 levels [EQ-5D-5L]).

The ERG considered that the company had successfully identified the available clinical outcomes and safety evidence for patients receiving venetoclax in the treatment of R/R CLL. Within the limits of their study design, the three included studies were judged to be of good quality. However, in the context of the STA decision problem, the ERG found what it considered to be serious deficiencies in the submitted evidence:

1. None of the included studies enrolled a population matching the specifications detailed in the licensed indications. In particular, patients’ status regarding previous chemoimmunotherapy was unclear, studies M12-175 and M13-982 failed to satisfy requirements about receiving or being unsuited for previous BCRi therapy, and studies M14-032 and M12-175 failed to satisfy a licensed specification for the del(17p)/TP53 chromosomal abnormality. This resulted in the company undertaking an ad hoc combination of study subgroups in order to develop the outcome estimates required to implement the cost-effectiveness analysis for the two indications.
2. No comparator evidence was available from the studies and the submitted clinical effectiveness section did not identify any other sources of such evidence (this was undertaken in the cost-effectiveness submission). As a consequence, estimates of effectiveness (venetoclax vs. comparator) were restricted to the cost-effectiveness section of the submission.
3. The studies were relatively small and of relatively short duration, resulting in substantial uncertainties associated with venetoclax outcomes, in particular in the time-to-event outcomes (OS and PFS) needed for cost-effectiveness analysis.
4. Almost all outcome results were designated AIC, and the ERG was unable to check the consistency of the data that were presented because it had not been published in peer review; there was an inevitable lack of transparency.

### Cost-Effectiveness Evidence

The submission provided a de novo partitioned-survival economic model comparing venetoclax with best supportive care (BSC) or with palliative care (PC). The model had three health states: pre-progression, post-progression and dead. The model operated a 28-day cycle length and had a lifetime horizon. The ERG judged the model structure to be appropriate to the available evidence for the decision problem—it was logical and appeared to capture two important features of the disease (PFS and OS); the cycle length (28 days) was sufficiently short to allow accurate modelling of changes over short time periods. The perspective, time horizon and discount rates followed NICE recommendations, and were appropriate to the decision problem.

The populations modelled in the submission were as follows: Indication 1: Patients with R/R CLL who had the del(17p)/TP53 aberration and whose disease had progressed after treatment with a BCRi; Indication 2: Patients with R/R CLL who lacked the del(17p)/TP53 aberration and whose disease had progressed after treatment with both chemoimmunotherapy and a BCRi. Patients in the venetoclax and BSC arms started in the progression-free health state, while patients in the PC arm started in the progressed disease health state.

Quality-of-life values for the progression-free health state were sourced using EQ-5D-5L from two venetoclax studies [3, 4] and a systematic review on health-related quality of life conducted by the company. Post-progression utility values were obtained from the systematic review. In their base-case, the company assumed that the utility value for del(17p)/TP53 and non-del(17p)/TP53 patients was the same and did not apply a quality-of-life decrement associated with adverse events.

Considerable challenges were faced in populating the model. These stemmed from the non-comparative nature of the venetoclax studies, the lack of conformity in their populations to the licensed indications, their small size, and their short duration relative to the model time horizon. The submission attempted to manage these difficulties, First, by pooling studies or study subgroups to increase patient numbers and produce populations conforming more closely to the licensed requirements; second, by using Weibull models for PFS and OS to extrapolate to the lifetime horizon and to generate transition probabilities between health states; and third, by identifying what was considered appropriate BSC and PC comparator populations that provided adequate PFS and OS data. Although statistical uncertainty may be reduced by pooling, the clinical and statistical validity of the simple combination of study patients may be questioned; however, it is acknowledged that because of the mixed populations in these small venetoclax trials, pooling may be expedient for the economic analysis.

To populate the venetoclax arm for indication 1, the submission pooled the del(17p)/TP53 subgroups from M12-175 and M14-032 with patients from the M13-982 study (all of whom carried the del(17p)/TP53 aberration). The resulting pooled population comprised only approximately half of the patients who had experienced previous BCRi therapy; no information was available regarding unsuitability for BCRi treatment, therefore the pooled population failed to fully meet the licensed requirements for indication 1. Weibull parametric models for OS and PFS were derived using the pooled population.

**Fig. 1:**
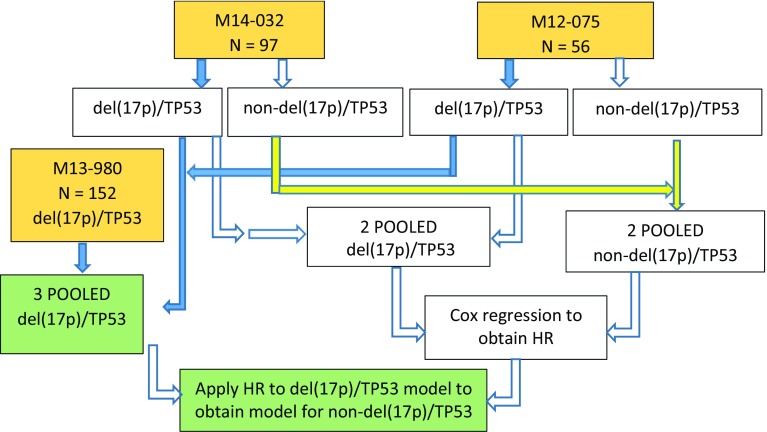
Company submission pooling procedures. *del(17p)* with 17p deletion, *non-del(17p)* without 17p deletion, *HR* hazard ratio, *N* sample size

To populate the venetoclax arm for indication 2, the submission derived OS and PFS hazard ratios (HRs) comparing non-del(17p)/TP53 with del(17p)/TP53, and applied these to the Weibull models of OS and PFS for indication 1 so as to generate Weibull models of OS and PFS for indication 2. To obtain the required HRs, the submission pooled del(17p)/TP53 patients from M14-032 with those from M12-175 to get a del(17p)/TP53 population, and pooled non-del(17p)/TP53 patients from M14-032 and M12-175 to get a non-del(17p)/TP53 population. These pooled groups were compared as if they were two arms of a single study, using Cox regression to obtain the required HRs. The company pooling procedures are summarised in Fig. 1.

The resulting OS and PFS Weibull models for indication 2 failed to fully meet the licensed requirements for indication 2 in terms of BCRi prior therapy since they are contingent on the curves generated for indication 1, which were derived from a population that substantially had not received prior BCRi therapy. Furthermore, the analysis for indication 2 involves some double counting of patients since the del(17p)/TP53 patients from the M12-175 and M14-032 studies contribute not only to the indication 1 curve but also to the HR estimation modifying it.

Rather than combine subgroups of patients from M14-032 and M12-175 and then use the combinations as though each was a single study, the ERG’s preferred procedure was to obtain an HR [non-del(17p)/TP53 vs. del(17p)/TP53] for each study and then pool the estimates using standard random effects meta-analysis. This produced a smaller HR for OS than the company’s method, and was therefore less favourable to venetoclax for non-del(17p)/TP53 OS relative to OS of any comparator.

The company applied the HRs for Weibull models of OS and PFS incorrectly. This was because the HRs were raised to the power of the shape parameter before being used as a multiplier for the Weibull scale parameter (see Supplementary Appendix). Since the shape parameters were greater than unity, the effect of this error was to inflate the performance of venetoclax for OS and PFS in indication 2; however, the effect was small because shape parameters were only slightly larger than unity and the HRs were not large. Because the submission adopted the same procedure for OS and PFS in their BSC comparator, and because the shape parameters were erroneously large for BSC (see Supplementary Appendix), the inflation of OS and PFS in the company’s BSC comparator for indication 2 was more substantial.

For the BSC comparator arm, the submission selected the placebo + rituximab arm from ‘study 116’, a double-blind, randomised controlled trial comparing idelalisib + rituximab versus placebo + rituximab [6]. Weibull regression data for OS and PFS were taken from the idelalisib STA submitted by Gilead to NICE [7]; this allowed the use of more mature data than that available in the published version of the study [6]. The ERG considered the use of the study 116 rituximab arm to be inappropriate and inconsistent with the licensed indication for venetoclax, because these patients had neither not responded to BCRi therapy, since they had not received it, nor were they judged inappropriate for BCRi therapy since they were eligible to be randomised to idelalisib. Another difficulty was the requirement for a technical correction to the survival analysis because of the substantial crossover in the rituximab arm to idelalisib treatment. The ERG considered that post-progression survival of patients from the idelalisib arm of study 116 provided a more appropriate representation of BSC since these patients had not responded to BCRi therapy and were thus more consistent with the venetoclax licensed indications.

To generate Weibull models of OS and PFS in BSC patients with the del(17p)/TP53) abnormality (indication 1), the company made use of Weibull regression parameters available from the idelalisib STA submission, which included regression values for 17p deletion status and for IGVH status (see Supplementary Appendix) [7]. In the ERG‘s opinion, the Weibull shape parameter was applied incorrectly (see Supplementary Appendix). To generate the Weibull model’s OS and PFS for BSC patients lacking the del(17p)/TP53) abnormality (indication 2), the submission applied the HRs it had derived for the comparison of non-del(17p)/TP53) versus del(17p)/TP53) in venetoclax patients. In the ERG’s opinion, it is questionable that the same HRs would hold across treatments with different mechanisms of action. Since the idelalisib submission provided the Weibull regression values for 17p deletion status, the ERG’s preferred modelling of the non-17p deletion BSC arm employed this parameter rather than an HR developed under venetoclax treatment, as used by AbbVie.

For the PC comparator the company submission proposed patients and data from the UK CLL Forum [8]. The selected PC population received no active intervention, and, although older, they matched reasonably well with the venetoclax study populations on known variables in the company submission; important prognostic variables identified by the CLL IPI study were not recorded (IGVH mutation status, microglobulin, and disease stage). The proposed PC survival data strongly suggested the presence of two quite distinct populations with a very different risk of death. The company was unable to find a satisfactory parametric fit for PC survival data and was unable to model the two indications separately. In the opinion of the ERG, the problematical construction of the PC population, the likelihood that two distinct subgroups were present, the lack of a good parametric fit for model extrapolation, the lack of correspondence between survival of the PC group and that of BCRi failures in other studies, and the inability to distinguish between non-del(17p)/TP53 and del(17p)/TP53 patients, rendered economic analysis of this PC comparator unsustainable. Furthermore, the company submission assumed BSC and PC to be competing treatments, whereas the ERG considers that BSC and PC would represent pathways of care for different (although overlapping) patient populations.

The costs of venetoclax treatment were provided by the company. Venetoclax treatment was assumed to continue until disease progression or toxicity, with the treatment effect persisting for up to 20 years. For BSC, patients received treatment (rituximab or rituximab + high-dose methylprednisolone) for six cycles only, and for patients in the PC arm, there was no active treatment. These costs were obtained from the British National Formulary [9]. The model also included costs for adverse events, routine care and terminal care, and for treatment of tumour lysis syndrome with rasburicase. All costs were presented in 2014/2015 prices.

The company’s revised base-case economic analysis indicated that venetoclax provided additional quality-adjusted life-years (QALYs), but at an additional cost. For del(17p)/TP53 patients whose disease had progressed after a BCRi, or for whom a BCRi was unsuitable, the incremental cost-effectiveness ratio (ICER) was £39,940 per QALY gained, and for non-del(17p)/TP53 patients whose disease had progressed after both chemoimmunotherapy and a BCRi, the ICER was £47,370 per QALY gained. The deterministic ICERs for venetoclax compared with BSC exceeded the usual norms accepted by NICE as suitable for reimbursement (£20,000–£30,000/QALY). In sensitivity analyses, these estimates were most influenced by the modelling of PFS and OS for indication 1, and by the HRs [for del(17p)/TP53 vs. non-del(17p)/TP53] for indication 2.

In critiquing the company‘s economic analysis, the ERG introduced several changes to the model inputs. The major change implemented by the ERG was to abandon the company models of OS and PFS for BSC based on the rituximab arm of study 116 and to instead substitute the post-progression survival of the idelalisib + rituximab arm of study 116. For the PC comparator, the ERG considered that the data used in the company submission had too many deficiencies for it to sustain a reasonable comparison for use in cost-effectiveness analysis. Additional changes involved correcting the application of HRs, correcting the starting age and proportion of males for patients with non-del(17p)/TP53, reducing the utility value for the PFS health state, and including disutility values for adverse events. When the ERG’s preferred inputs were applied to the company’s base-case, the resulting ICERs for venetoclax versus BSC for indications 1 (£55,476/QALY gained) and 2 (£77,779/QALY gained) increased substantially. In sensitivity analysis, the ERG employed post-progression survival after failure of ibrutinib as the BSC comparator, taking data from Jansen’s submission to NICE for the STA ‘Ibrutinib for treating relapsed or refractory CLL’ [10]. Again, this delivered an ICER (£61,120/QALY gained) higher than the company’s base-case ICER for indication 1.

### Conclusions of the ERG Review

The three trials sourced for evidence in the company submission included patients who did not meet the decision problem. Evidence that better met the decision problem was based on *post hoc* subgroup combinations from single-arm studies with heterogeneous populations. The absence of direct or formal indirect comparisons means that the true treatment benefit of venetoclax is uncertain. The company submission selected one arm of an unrelated RCT to approximate the comparator of BSC, however the ERG considers that the population in that arm is unsuitable as a comparator group. Caution is therefore necessary in the interpretation of the submitted results. Even though the company’s economic model was appropriate, its shortcomings were mainly due to the lack of an appropriate comparator(s). The BSC group chosen in the company submission was a misfit for the decision problem and for the licensed indications. The PC comparator data used in the company submission had too many deficiencies for it to sustain a reasonable comparison for use in cost-effectiveness analysis.

## Development of National Institute for Health and Care Excellence Guidance

In developing guidance, the NICE Appraisal Committee’s preliminary recommendation was that venetoclax used within its licensed indication should not be recommended for use in the NHS. With regard to the BSC comparator, the committee suggested that the comparator population selected by the ERG more closely matched the population that would be offered venetoclax than that selected in the company submission. However, the committee expressed concern that whatever the source of comparator data used, the comparisons would be naive and potentially subject to bias. The committee preferred the ERG’s utility value for the PFS health state. The company submission value based on pooled data from the venetoclax trials was considered implausible since patients with CLL would not be expected to have a higher quality of life than people of the same age without disease. The committee concluded that it preferred the ERG’s base-case, which included disutility for adverse events, and updated the costs of some adverse events included in the company submission.

In response to the preliminary recommendation, the company submitted new analyses. In a second appraisal meeting, the committee concluded there were large uncertainties around the clinical effectiveness of venetoclax and BSC, and that under the committee’s preferred assumptions, the ICERs were higher than those generally considered cost effective, even when end-of-life criteria were taken into account. In their final guidance published in November 2017 [11] NICE recommended venetoclax for use with the Cancer Drugs Fund for patients with indications 1 and 2.

## Electronic supplementary material

Below is the link to the electronic supplementary material.

Supplementary material 1 (DOCX 187 kb)
