# Correction to: Venetoclax for Treating Chronic Lymphocytic Leukaemia: An Evidence Review Group Perspective of a NICE Single Technology Appraisal

**DOI:** 10.1007/s40273-019-00773-w

**Published:** 2019-02-19

**Authors:** Hema Mistry, Chidozie Nduka, Martin Connock, Jill Colquitt, Theodoros Mantopoulos, Emma Loveman, Renata Walewska, James Mason

**Affiliations:** 1 Warwick Evidence, Warwick Medical School, University of Warwick, Gibbet Hill Road, Coventry, CV4 7AL UK; 2 Effective Evidence LLP, 26 The Curve, Waterlooville, Hampshire PO8 9SE UK; 3 The Royal Bournemouth Hospital NHS Foundation Trust, Castle Lane East, Bournemouth, BH7 7DW UK

## Correction to: PharmacoEconomics (2018) 36:399–406 10.1007/s40273-017-0599-9

The Open Access license, which previously read:

**Open Access:** This article is distributed under the terms of the Creative Commons Attribution-NonCommercial 4.0 International License (http://creativecommons.org/licenses/by-nc/4.0/), which permits any noncommercial use, distribution, and reproduction in any medium, provided you give appropriate credit to the original author(s) and the source, provide a link to the Creative Commons license, and indicate if changes were made.

Should read:

**Open Access:** This article is distributed under the terms of the Creative Commons Attribution 4.0 International License (http://creativecommons.org/licenses/by/4.0/), which permits unrestricted use, distribution, and reproduction in any medium, provided you give appropriate credit to the original author(s) and the source, provide a link to the Creative Commons license, and indicate if changes were made.

The original article was corrected.

