# Supplementary material for: Venetoclax for Treating Chronic Lymphocytic Leukaemia: An Evidence Review Group Perspective of a NICE Single Technology Appraisal
Source: Pharmacoeconomics. 2017 Dec 8;36(4):399–406. doi: 10.1007/s40273-017-0599-9 (PMC5840199; doi:10.1007/s40273-017-0599-9)
Supplement: Supplementary file 1 — Supplementary material 1 (DOCX 187 kb) [file 40273_2017_599_MOESM1_ESM.docx]

**APPENDIX**

1. **Weibull parameterisation for venetoclax recipients**

The company’s submitted cost-effectiveness model employed the following parameterisation for Weibull models of OS and PFS for del(17p)/TP53 venetoclax recipients:

S = EXP (- (((T x 1/SCd)**^sh^**

Where T = time, SCd = scale parameter for del(17p)/TP53 patients and sh = shape parameter.

To obtain Weibull models of OS and PFS for non-del(17p)/TP53 patients, proportional hazards was assumed for del(17p)/TP53 positive versus non-del(17p)/TP53, and the parametrisation was modified using a hazard ratio (for non-del(17p)/TP53 versus del(17p)/TP53) as follows:

S = EXP (- (((T x 1/SCd x HR)**^sh^**

In Weibull models, the scale parameter is multiplied by the HR, whereas in the company’s parameterisation the scale parameter was multiplied the HR raised to the power of the shape parameter; the company’s model was therefore incorrect. The ERG nullified this error by simply raising the HR to the power of 1/sh, before entering the modified value into the company’s equation. In clarification the company offered an alternative method to correct for the error.

The company’s estimated HRs for the comparison non-del(17p)/TP53 versus del(17p)/TP53 were 1.908 and 1.708 for OS and PFS respectively, and the shape parameters were 1.174 for OS and 1.126 for PFS. Thus in the company models the scale was multiplied by inflated HR values (1.908^1.174^ = 2.135 for OS, and 1.708^1.126^ = 1.827 for PFS). Because the shape parameters were near unity the erroneous values used are close to the correct values and therefor the influence of the error on the resulting survival models in these instances was relatively minor.

1. **Weibull parameterisation for BSC recipients**

In order to model OS and PFS for del(17p)/TP53 BSC recipients the company employed data from an STA submitted by Gilead to NICE.^1^ The data used came from Table 88 of the Gilead submission which provided Weibull regression values for OS and PFS for each arm of the “study 116” RCT that compared placebo + rituximab versus idelalisib + rituximab. The venetoclax submission employed the regression parameters from Table 88 for the placebo + rituximab arm which are summarised in the Table 2 below and these values can be compared with the presentation in Table 56 of the company submission.

**Table 2: Summary of variables used for parameterisation**

| Gilead Table 88 OS Regression Value | AbbVie Table 56 OS value | Gilead Table 88 PFS Regression Value | AbbVie Table 56 PFS value |  | Proportion of patients (AbbVie company submission Table 56) |
| --- | --- | --- | --- | --- | --- |
| Intercept 3.04 | 3.04 | Intercept 2.08 | 2.08 |  |  |
| DEL -0.61 | -0.61 | DEL -0.39 | -0.39 |  | 1 |
| IGVH 0.57 | 0.57 | IGVH 0.37 | 0.37 |  | 0.231 |
| Shape 0.75 | 2.12§ | Shape 0.61 | 1.84§§ |  |  |
| § EXP (0.75) =2.12. §§ EXP (0.61) = 1.84 | | | |  |  |

In comparing Gilead Table 88 regression values with the venetoclax submission Table 56 values, it is evident that AbbVie derived their Weibull shape parameter by taking the exponential of the Gilead Table 88 regression value. The ERG considers this to be an error and that the correct Weibull shape parameter is obtained by taking the reciprocal of the Gilead Table 88 regression value. Thus the Weibull shape parameter for OS should be 1.333 (=1/0.75) rather than 2.12, and for PFS the Weibull shape should be 1.64 (=1/0.61) rather than 1.84.

Table 56 of the venetoclax submission derived the OS scale parameter as follows:

SCd = EXP (3.04 + (-0.61 X 1) + (0.57 X 0.231)) = 12.96;

The PFS scale parameter is derived as:

SCd = EXP (2.08 + (-0.39 X 1) + (0.37 X 0.231)) = 5.90.

The parameterisations for OS and for PFS then become:

OS = EXP (- (((T x 1/12.96)^sh^ where **sh** is 1.333 according to the ERG or 2.12 according to AbbVie

PFS = EXP (- (((T x 1/5.90)**^sh^** where **sh** is 1.64 according to the ERG or 1.84 according to AbbVie.

To test whether the ERG interpretation of the Gilead Table 88 regression value is correct the ERG attempted to reproduce the Gilead OS Weibull models for each arm of study 116 presented as Figure 26 in the Gilead submission. The requisite data from the Gilead submission are summarised in Table 3.

**Table 3: Summary of parameter values used for the survival curves**

| **Idelalisib arm Table 88 regression values** | **Patient proportions in**  **idelalisib arm** | **Rituximab arm Table 88 regression values** | **Proportions in**  **rituximab arm** |
| --- | --- | --- | --- |
| intercept 4.20 |  | intercept 3.04 |  |
| del -0.45 | 0.4182 | del -0.61 | 0.4455 |
| IGHV 0.43 | 0.1727 | IGHV 0.57 | 0.1545 |
| shape 0.77 |  | shape 0.75 |  |
| scale = EXP(4.2+(-0.45x0.4182)+(0.43x0.127)) = 59.51  shape = exp(0.77)=2.16 OR shape = 1/0.77 = 1.30  OS=EXP(T x 1/59.51)^2.16 or 1.30^ | | scale = EXP(3.04+(-0.61x0.4455)+(0.57x0.1545)) = 17.40  shape = exp(0.75)=2.12 OR shape = 1/0.75 = 1.33  OS=EXP(T x 1/17.40)^2.12 or 1.333^ | |

The resulting plots are shown in Figure 2.

**Figure 2: Weibull models of OS in study 116.**

R = placebo + rituximab arm; IR = idelalisib + rituximab arm. Dashed lines use the venetoclax submission’s derivation of shape parameters the dotted lines use the ERG’s derivation of shape parameters.

When these plots were overlaid onto Figure 21 of the Gilead submission, the ERG plots exactly followed the Gilead plots whereas the plots using the AbbVie interpretation of the Gilead shape regression value generated considerably different (more sigmoid) survival curves. The ERG assumes that the Gilead submission used its own regression analyses correctly and therefore that the ERG interpretation of the shape parameter is more likely correct than the AbbVie interpretation.

The use of the incorrect shape parameters leads to inaccurate estimation of OS and PFS in BSC recipients for both del(17p)/TP53 and non-del(17p)/TP53 subgroups in the company’s base-case economic analysis. To obtain the OS and PFS for non-del(17p)/TP53 recipients of BSC the company applied the same HRs as were used for venetoclax recipients and applied these to the BSC Weibull models for del(17p)/TP53 patients. As with the company’s venetoclax analyses the HRs were applied incorrectly (that is they were incorrectly raised to the power of the shape parameter (which for BSC was also incorrect as described above).

**References**

1. National Institute for Health and Care Excellence. *Idelalisib for treating chronic lymphocytic leukaemia: Technology appraisal guidance [TA359]*. 2015. URL: <https://www.nice.org.uk/guidance/ta359> (Accessed 11/10/2016).
